# Weekly versus every-three-weeks platinum-based chemoradiation regimens for head and neck cancer

**DOI:** 10.1186/s40463-016-0175-x

**Published:** 2016-11-24

**Authors:** James M. Melotek, Benjamin T. Cooper, Matthew Koshy, Joshua S. Silverman, Michael T. Spiotto

**Affiliations:** 1 Department of Radiation and Cellular Oncology, University of Chicago, KCBD 6142, 900 E. 57th St, Chicago, IL 60637 USA; 2 Department of Radiation Oncology, New York University, New York, NY USA; 3 Department of Radiation Oncology, University of Illinois at Chicago, Chicago, IL USA

**Keywords:** Head and neck neoplasm, Concurrent chemoradiotherapy, Acute kidney injury, Cisplatin, Carboplatin

## Abstract

**Background:**

The majority of chemoradiation (CRT) trials for locally advanced head and neck squamous cell carcinoma (HNSCC) have relied on platinum-based chemotherapy regimens administered every-3-weeks. However, given the increased utilization of weekly platinum regimens, it remains unclear how different chemotherapy schedules compare regarding efficacy and toxicity.

**Methods:**

We retrospectively identified 212 patients with HNSCC who were treated at a single academic medical center with concurrent platinum-based CRT given weekly (*N* = 68) or every-three-weeks (*N* = 144). JMP version 10 (SAS Institute) was used for statistical analysis. Discrete variables were compared with the chi-square test and differences in the medians were assessed using the Wilcoxon test. Survival curves were constructed using the Kaplan-Meier method and significance was assessed using the log rank test. For univariate analysis and multivariate analysis, we used Cox proportional hazard or logistic regression models to compare differences in survival or differences in categorical variables, respectively.

**Results:**

Patients receiving weekly platinum regimens were more likely to be older (median age 61.4 vs. 55.5 y; *P* < .001), have high or very high Charlson comorbidity index (45.6% vs. 27.8%; *P* = .01), and receive carboplatin-based chemotherapy (6.3% vs. 76.5%; *P* < .001). Weekly and every-3-week platinum regimens had similar locoregional control (HR 1.10; 95% CI 0.63–1.88; *P* = .72), progression-free survival (HR 1.13; 95% CI 0.75–1.69; *P* = .55), and overall survival (HR 1.11; 95% CI 0.64–1.86; *P* = .71). Every-3-weeks platinum regimens were associated with increased days of hospitalization (median: 3 days vs. 0 days; *P* = .03) and acute kidney injury (AKI) during radiotherapy (50.0% vs. 22.1%; *P* < .001). On multivariate analysis, AKI was significantly associated with every-3-weeks regimens (OR: 24.38; 95% CI 3.00–198.03; *P* = .003) and high comorbidity scores (OR: 2.74; 95% CI 2.15–5.99; *P* = .01).

**Conclusions:**

Our results suggest that every-3-weeks and weekly platinum-containing CRT regimens have similar disease control but weekly platinum regimens are associated with less acute toxicity.

**Electronic supplementary material:**

The online version of this article (doi:10.1186/s40463-016-0175-x) contains supplementary material, which is available to authorized users.

## Background

In patients with locally advanced head and neck squamous cell carcinoma (HNSCC), chemoradiation (CRT) improves locoregional control (LRC) and overall survival (OS) compared to radiotherapy (RT) alone [1]. Typically, CRT schedules use platinum-based regimens given every three weeks [2–6]. The Head and Neck Intergroup trial and RTOG 91-11 observed that concurrent CRT with every-3-weeks cisplatin improved LRC and OS for unresectable HNSCC and resectable laryngeal cancers, respectively [2, 4]. Other trials have demonstrated improved outcomes using every-3-weeks platinum-based chemotherapy alone or combined with 5-fluorouracil and/or other drugs during RT [3, 5, 6]. Due to the prevalence of randomized studies, cisplatin at a dose of 100 mg/m^2^ given every three weeks remains the recommended systemic therapy option during CRT [7]. However, the addition of this chemotherapy regimen to RT is often associated with significantly increased toxicities including stomatitis, nausea/vomiting, and myelosuppression. For example, rates of grade ≥ 3 stomatitis and myelosuppression were 73% and 81% with CRT compared to 42% and 5% with RT alone in RTOG 91-11 [4]. Thus, there remains a critical need to maintain the efficacy of CRT while minimizing its toxicity.

Several groups have investigated the use of a weekly platinum-based chemotherapy schedule during RT to reduce toxicity [8–11]. Data regarding this approach are limited and conflicting. Chan et al. demonstrated an overall survival benefit with the addition of weekly cisplatin to RT in nasopharyngeal carcinoma [12]. However, LRC was not improved, contrary to expectations with the use of radiosensitizing doses of chemotherapy. Additional support for weekly platinum CRT regimens has been extrapolated from two cervical carcinoma trials demonstrating improved OS and progression-free survival (PFS) with weekly cisplatin [13, 14]. However, another trial by the National Cancer Institute of Canada observed no difference in outcomes between RT and CRT with weekly platinum [15]. In the setting of unresectable head and neck cancer, a joint trial performed by the RTOG and ECOG showed no benefit in LRC or OS with the addition of weekly low dose cisplatin at 20 mg/m^2^ to RT [16]. Furthermore, retrospective or prospective data comparing the efficacy and toxicity of weekly versus every-3-weeks platinum-based CRT are lacking. We therefore sought to compare the outcomes of patients with HNSCC treated with platinum-based CRT at a single institution using either a weekly or every-3-weeks chemotherapy schedule.

## Methods

### Study population

Patients were treated at the University of Illinois Medical Center at Chicago between 1992 and 2012. This study was approved by the University of Illinois Medical Center IRB protocol 2011-1075 in accordance with the ethical standards of the responsible committee on human experimentation and with the Helsinki Declaration of 1999, as revised in 2000. The University of Illinois at Chicago IRB waived informed consent given that this study used preexisting medical records and obtaining informed consent on all patients would be impractical given the associated time and cost. Patient data was anonymized and de-identified prior to analysis. We identified 212 consecutive patients with HNSCC from a retrospective database who were treated with concurrent platinum-based CRT given weekly or every-three-weeks. 144 patients received every-3-weeks chemotherapy and 68 patients received weekly chemotherapy. Patients were excluded who did not have documented chemotherapy schedules or received a combination of weekly and every-3-weeks chemotherapy. Of patients receiving every-3-weeks chemotherapy, 3 patients initially received cisplatin for the first 1-2 cycles and were subsequently switched to carboplatin. The anonymized and de-identified dataset is available for review as Additional file 1.

### Variables

Data was collected from all available physical and electronic medical records. All patients were included in this analysis regardless of treatment compliance. During RT, acute toxicities were recorded during weekly on-treatment visits. We approximated comorbidity burden using a modified Charlson Comorbidity Index [17] and performance status using the Karnofsky Performance Status (KPS) [18]. Staging was categorized using the American Joint Committee on Cancer staging system used at the time of diagnosis. We defined RT delay as RT courses that were completed 3 days or longer than the anticipated finish date and RT truncations as RT courses that did not achieve the prescribed radiation dose. Chemotherapy modifications included treatments that did not achieve 3 cycles of every-3-weeks platinum chemotherapy or 6 cycles of weekly platinum chemotherapy, chemotherapy dose reductions, or changes in chemotherapy drugs. We defined the length and number of hospitalizations based on the discharge summary. Creatinine (Cr) values were obtained from patient records and were measured at the start of therapy, during RT at one to two week intervals, and at regular follow-up visits. We defined acute kidney injury (AKI) as a peak Cr concentration during radiotherapy greater than or equal to 0.3 mg/dL over pretreatment Cr levels as it meets one of the three Acute Kidney Injury Network (AKIN) criteria for AKI [19] and Cr increments as small as 0.3–0.5 mg/dL have been associated with increased mortality [20]. Time to LRC, PFS, and OS were determined from last date of RT. Patterns of failure were determined as the first failure with any component of local, regional or distant recurrence, respectively. PFS was calculated as the time to any failure or death. OS was calculated as the time to death.

### Statistical analysis

We used JMP version 10 (SAS Institute) to perform statistical analysis using two-sided tests and defining significance as *P* < .05. Discrete variables were compared with the chi-square test and differences in the medians were assessed using the Wilcoxon test. Survival curves were plotted using the Kaplan-Meier method and significance was assessed using the log rank test. For univariate analysis (UVA) and multivariate analysis (MVA), we used Cox proportional hazard or logistic regression models to compare differences in survival or differences in categorical variables, respectively. Censoring is assumed to be non-informative. Variables with *P* value < .1 on UVA were included on MVA.

## Results

### Population, tumor, and treatment characteristics

As shown in Table 1, median follow-up was not significantly different between groups (21.1 months for every-3-weeks vs. 23.7 months for weekly chemotherapy; *P* = .40). Patients receiving every-3-weeks chemotherapy were younger (55.5y vs. 61.4y; *P* < .001) and had lower comorbidity scores (27.8% vs. 45.6% with high comorbidity index; *P* = .01). There was no difference in gender, performance status, smoking or alcohol use, primary site, tumor stage, or nodal stage. In an analysis limited to patients who received either every-3-weeks cisplatin or weekly carboplatin also shown in Table 1, patients receiving every-3-weeks cisplatin were younger (55.4y vs. 61.9y; *P* < .001), more likely to be male (81.2% vs. 67.3%; *P* = .04), and had lower comorbidity scores (26.1% vs. 48.1% with high comorbidity index; *P* < .01). As shown in Table 2, patients receiving weekly chemotherapy were more often treated in the post-operative setting (44.1% vs 30.6%; *P* = .05) and received carboplatin chemotherapy (76.5% vs 6.3%; *P* < .001). There was no difference in receipt of induction chemotherapy, post-radiation lymph node dissection, alterations in RT course, RT technique (3D-conformal vs. intensity-modulated RT), or chemotherapy dose modification. In an analysis limited to patients who received either every-3-weeks cisplatin or weekly carboplatin also shown in Table 2, there were no significant differences in treatment characteristics other than the chemotherapy agent delivered. Patients in the every-3-weeks cisplatin group received a median cumulative dose of 200 mg/m^2^ (interquartile range 200 mg/m^2^ – 300 mg/m^2^).

**Table 1:** Patient characteristics

	Every-3-weeks platinum	Weekly platinum	*P-*value	Every-3-weeks cisplatinum	Weekly carboplatinum	*P-*value
	(*N* = 144)	(*N* = 68)		(*N* = 138)	(*N* = 52)	
Median age (years)	55.5	61.4	<.001	55.4	61.9	<.001
(IQR)	(48.1–62.0)	(51.7–71.6)		(47.6–61.9)	(52.1–71.5)	
Median follow-up (months)	21.1	23.7	.40	21.1	23.7	.70
(IQR)	(12.2–59.0)	(11.8–38.3)		(12.1–56.0)	(11.3–45.1)	
Gender			.16			.04
Male	116 (80.6%)	49 (72.1%)		112 (81.2%)	35 (67.3%)	
Female	28 (19.4%)	19 (27.9%)		26 (18.8%)	17 (32.7%)	
KPS			.30			.68
≥ 70	120 (83.3%)	51 (75.0%)		116 (84.1%)	41 (78.9%)	
< 70	9 (6.3%)	5 (2.4%)		9 (6.5%)	4 (7.7%)	
Not stated	15 (10.4%)	12 (17.7%)		13 (9.4%)	7 (13.5%)	
Comorbidity index			.01			<.01
Medium	104 (72.2%)	37 (54.4%)		102 (73.9%)	27 (51.9%)	
High	40 (27.8%)	31 (45.6%)		36 (26.1%)	25 (48.1%)	
Stage			.34			.33
I	0 (0.0%)	1 (1.5%)		0 (0.0%)	1 (1.9%)	
II	10 (6.9%)	3 (4.4%)		9 (6.6%)	2 (3.9%)	
III	33 (22.9%)	19 (27.9%)		31 (22.6%)	10 (19.2%)	
IV	101 (70.1%)	45 (66.2%)		97 (70.8%)	39 (75.0%)	
Alcohol history			.39			.35
≥ 2 drinks/day	76 (52.8%)	33 (48.5%)		73 (52.9%)	25 (48.1%)	
< 2 drinks/day	36 (25.0%)	21 (30.9%)		35 (25.4%)	17 (32.7%)	
Not stated	32 (22.2%)	14 (20.6%)		30 (21.7%)	10 (19.2%)	
Tobacco history			.47			.84
> 10 pack-years	106 (73.6%)	48 (70.6%)		101 (73.2%)	38 (73.1%)	
≤ 10 pack-years	33 (22.9%)	19 (27.9%)		32 (23.2%)	13 (25.0%)	
Not stated	5 (3.5%)	1 (1.5%)		5 (3.6%)	1 (1.9%)	
Primary site			.83			.68
Hypopharynx	5 (3.5%)	4 (5.9%)		5 (3.6%)	3 (5.8%)	
Larynx	31 (21.5%)	12 (17.7%)		29 (21.0%)	9 (17.3%)	
Nasopharynx	14 (9.7%)	5 (7.4%)		14 (10.1%)	3 (5.8%)	
Oral Cavity	38 (26.4%)	16 (23.5%)		36 (26.1%)	11 (21.2%)	
Oropharynx	39 (27.1%)	20 (29.4%)		38 (27.5%)	17 (32.7%)	
Other	17 (11.8%)	11 (16.2%)		16 (11.6%)	9 (17.3%)	
Tumor Stage			.27			.10
T0-2	44 (30.6%)	26 (38.2%)		41 (29.7%)	22 (42.3%)	
T3-4b	100 (69.4%)	42 (61.8%)		97 (70.3%)	30 (57.7%)	
Nodal Stage			.14			.68
N0-2a	69 (47.9%)	40 (58.8%)		67 (48.6%)	27 (51.9%)	
N2b-3	75 (52.1%)	28 (41.2%)		71 (51.5%)	25 (48.1%)	
P16 status			.11			.07
Positive	4 (2.8%)	0 (0.0%)		4 (2.9%)	0 (0.0%)	
Negative	50 (34.7%)	32 (47.1%)		49 (35.5%)	27 (51.9%)	
Not stated	90 (62.3%)	36 (52.9%)		85 (61.6%)	25 (48.1%)	

*Cr* creatinine, *IQR* interquartile range, *KPS* Karnofsky performance status

**Table 2:** Treatment characteristics

	Every-3-weeks platinum	Weekly platinum	*P-*value	Every-3-weeks cisplatinum	Weekly carboplatinum	*P-*value
	(*N* = 144)	(*N* = 68)		(*N* = 138)	(*N* = 52)	
RT timing			.05			.18
Post-operative	44 (30.6%)	30 (44.1%)		44 (31.9%)	22 (42.3%)	
Definitive	100 (69.4%)	38 (55.9%)		94 (68.1%)	30 (57.7%)	
Induction chemotherapy			.60			.59
Yes	41 (28.5%)	17 (25.0%)		37 (26.8%)	16 (30.8%)	
No	103 (71.5%)	51 (75.0%)		101 (73.2%)	36 (69.2%)	
Post-radiation lymph node dissection			.42			.47
Yes	19 (13.2%)	5 (7.4%)		18 (13.0%)	4 (7.7%)	
No	81 (56.3%)	33 (48.5%)		76 (55.1%)	26 (50.0%)	
Not stated	44 (30.6%)	30 (44.1%)		44 (31.9%)	22 (42.3%)	
RT technique			.57			0.68
3D-conformal	25 (17.4%)	14 (20.6%)		23 (16.7%)	10 (19.2%)	
IMRT	119 (82.6%)	54 (79.4%)		115 (83.3%)	42 (80.8%)	
Type of chemotherapy			<.001			<.001
Cisplatin	138 (95.8%)^a^	16 (23.5%)		138 (100.0%)	0 (0.0%)	
Carboplatin	9 (6.3%)	52 (76.5%)		0 (0.0%)	52 (100.0%)	
Alterations in RT course			.83			.90
None	82 (56.9%)	40 (58.8%)		78 (56.5%)	31 (59.6%)	
Delay	43 (29.9%)	21 (30.9%)		41 (29.7%)	15 (28.9%)	
Truncations	19 (13.2%)	7 (10.3%)		19 (13.8%)	6 (11.5%)	
Chemotherapy dose modification			.44			.55
Yes	76 (52.8%)	32 (47.1%)		73 (52.9%)	25 (48.1%)	
No	68 (47.2%)	36 (52.9%)		65 (47.1%)	27 (51.9%)	

*RT* radiotherapy, *IMRT* intensity modulated radiotherapy

^a^Three patients were treated with CDDP and switched to carboplatin. All were treated according to an every-3-weeks regimen

### Outcomes and toxicity

**Fig. 1:**
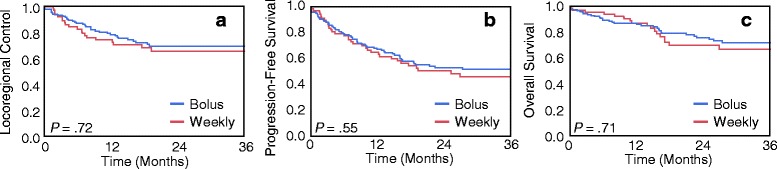
Kaplan-Meier curves for (**a**) locoregional control, (**b**) progression-free survival, and (**c**) overall survival in patients receiving weekly versus every-3-weeks chemoradiation regimens. The log rank test was used to assess for differences in outcomes

**Fig. 2:**
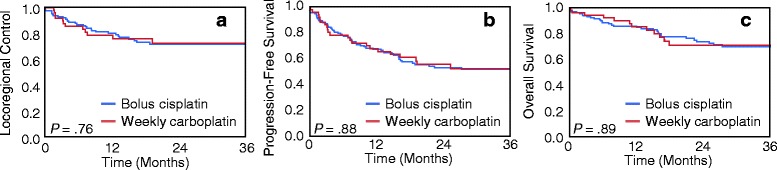
Kaplan-Meier curves for (**a**) locoregional control, (**b**) progression-free survival, and (**c**) overall survival in patients receiving weekly carboplatin versus every-3-weeks cisplatin chemoradiation regimens. The log rank test was used to assess for differences in outcomes

With median follow-up of 23.7 months for the entire cohort, 85 patients experienced disease progression (28 patients in the weekly chemotherapy group and 57 patients in the every-3-weeks chemotherapy group). The majority of failures were due to locoregional progression (20 patients in the weekly chemotherapy group and 38 patients in the every-3-weeks chemotherapy group). At the time of analysis, 63 patients had died (20 patients in the weekly chemotherapy group and 43 patients in the every-3-weeks chemotherapy group). As shown in Fig. 1, weekly chemotherapy in comparison to every-3-weeks chemotherapy was not associated with worse LRC (2y LRC ± SE 65.7 ± 6.4% vs. 69.7 ± 4.4%; HR 1.10; 95% CI 0.63–1.88; *P* = .72), PFS (2y PFS ± SE 50.7 ± 6.4% vs. 53.1 ± 4.6%; HR 1.13; 95% CI 0.75–1.69; *P* = .55), or OS (2y OS ± SE 69.9 ± 6.4% vs. 75.7 ± 4.0%; HR 1.11; 95% CI 0.64–1.86; *P* = .71). As shown in Fig. 2, weekly carboplatin in comparison to bolus cisplatin was not associated with worse LRC (2y LRC ± SE 72.7 ± 6.9% vs. 71.1 ± 4.5%; HR 0.90; 95% CI 0.45–1.70; *P* = .76), PFS (2y PFS ± SE 55.8 ± 7.4% vs. 53.3 ± 4.8%; HR 0.96; 95% CI 0.59–1.52; *P =* .88), or OS (2y OS ± SE 71.2 ± 7.2% vs. 74.6 ± 4.3%; HR 0.96; 95% CI 0.50–1.71; *P =* .89).

When only patients treated with definitive RT were analyzed, weekly chemotherapy in comparison to every-3-weeks chemotherapy was not associated with worse LRC (2y LRC ± SE 55.2 ± 9.6% vs. 61.9 ± 5.7%; HR 1.29; 95% CI 0.66–2.38; *P* = .43), PFS (2y PFS ± SE 39.8 ± 8.7% vs. 46.6 ± 5.5%; HR 1.42; 95% CI 0.86–2.29; *P* = .16), or OS (2y OS ± SE 66.8 ± 8.9% vs. 75.4 ± 5.1%; HR 1.58; 95% CI 0.78–3.02; *P* = .18). When only patients treated with adjuvant RT were analyzed, weekly administration of chemotherapy was also not associated with worse LRC (2y LRC ± SE 77.2 ± 8.3% vs. 87.6 ± 5.2%; HR 1.32; 95% CI 0.41–4.25; *P* = .63), PFS (2y PFS ± SE 63.1 ± 9.4% vs. 67.9 ± 7.5%; HR 1.00; 95% CI 0.46–2.11; *P* = 1.00), or OS (2y OS ± SE 74.0 ± 9.2% vs. 76.9 ± 6.9%; HR 0.82; 95% CI 0.30–2.04; *P* = .67).

On UVA (Table 3), every-three-weeks chemotherapy was associated with increased median total hospital days (3 vs. 0; *P* = .03) and AKI during RT (50.0% vs. 22.1%; *P* < .001). By contrast, receipt of every-three-weeks chemotherapy was not associated with acute toxicities such as weight loss, feeding tube during or after RT, tracheotomy dependence after RT, mucositis or dermatitis. On UVA limited to patients who received either every-3-weeks cisplatin or weekly carboplatin also shown in Table 3, every-3-weeks cisplatin was similarly associated with AKI during RT (49.3% vs. 28.9%; *P* < .01). On MVA (Table 4), AKI during RT was significantly associated with receipt of every-3-weeks chemotherapy (OR: 24.38; 95% CI 3.00–198.03; *P* = .003) and high comorbidity index (OR: 2.74; 95% CI 1.25–5.99; *P* = .01) but not with receipt of cisplatin chemotherapy, post-operative RT, or age. Only high comorbidity index was significantly associated with hospitalizations on MVA (OR: 2.08; 95% CI 1.05–4.21; *P* = .03).

**Table 3:** Toxicity

	Every-3-weeks platinum	Weekly platinum	*P-*value	Every-3-weeks cisplatinum	Weekly carboplatinum	*P*-value
	(*N* = 144)	(*N* = 68)		(*N* = 138)	(*N* = 52)	
Median total hospital days	3	0	.03	3	0	.08
(IQR)	(0–9)	(0–4)		(0–9)	(0–4)	
Hospitalizations			.04			.04
Yes	62 (43.1%)	28 (41.2%)		60 (43.5%)	21 (40.4%)	
No	49 (34.0%)	33 (48.5%)		46 (33.3%)	26 (50.0%)	
Not stated	33 (22.9%)	7 (10.3%)		32 (23.2%)	5 (9.6%)	
Feeding tube during RT			.11			.09
Yes	72 (50.0%)	26 (38.2%)		67 (48.6%)	18 (34.6%)	
No	72 (50.0%)	42 (61.8%)		71 (51.5%)	34 (65.4%)	
Weight loss >10%			.17			.06
Yes	72 (50.0%)	30 (44.1%)		71 (51.4%)	23 (44.2%)	
No	36 (25.0%)	24 (35.3%)		33 (23.9%)	21 (40.4%)	
Not stated	36 (25.0%)	14 (20.6%)		34 (24.6%)	8 (15.4%)	
Grade ≥ 3 mucositis			.47			.25
Yes	35 (24.3%)	19 (27.9%)		33 (23.9%)	17 (32.7%)	
No	85 (59.0%)	36 (52.9%)		83 (60.1%)	28 (53.8%)	
Not stated	24 (16.7%)	13 (19.1%)		22 (15.9%)	7 (13.5%)	
Grade ≥ 3 dermatitis			.54			.70
Yes	6 (4.2%)	4 (5.9%)		6 (4.3%)	3 (5.8%)	
No	115 (79.9%)	51 (75.0%)		111 (80.4%)	42 (80.8%)	
Not stated	23 (16.0%)	13 (19.1%)		21 (15.2%)	7 (13.5%)	
Feeding tube at last follow-up			.46			.15
Yes	43 (29.9%)	17 (25.0%)		41 (29.7%)	10 (19.2%)	
No	101 (70.1%)	51 (75.0%)		97 (70.3%)	42 (80.8%)	
Tracheotomy at last follow-up			.67			.21
Yes	20 (13.9%)	8 (11.8%)		20 (14.5%)	4 (7.7%)	
No	124 (86.1%)	60 (88.2%)		118 (85.5%)	48 (92.3%)	
AKI during RT			<.001			<.01
Yes	72 (50.0%)	15 (22.1%)		68 (49.3%)	15 (28.9%)	
No	40 (27.8%)	40 (58.8%)		39 (28.3%)	29 (55.8%)	
Not stated	32 (22.2%)	13 (19.1%)		31 (%)	8 (15.4%)	

*AKI* acute kidney injury, *IQR* interquartile range, *RT* radiotherapy

**Table 4:** Multivariate analysis for factors impacting toxicity

	Odds ratio (95% CI)	
	AKI during RT	Hospitalizations
High comorbidity index	2.74 (1.25–5.99)	2.08 (1.05–4.21)
*P* value	.01	.03
Every-3-weeks chemotherapy	24.38 (3.00–198.03)	1.16 (0.41–3.26)
*P* value	.003	.20
Post-operative RT	0.60 (0.29–1.24)	0.66 (0.34–1.25)
*P* value	.17	.20
Cisplatin	6.38 (0.78–52.31)	1.94 (0.67–5.78)
*P* value	.08	.22
Age ≥ 60	0.59 (0.29–1.22)	1.64 (0.85–3.23)
*P* value	.60	.14

*AKI* acute kidney injury, *CI* confidence interval, *RT* radiotherapy

## Discussion

In our cohort of patients with HNSCC treated with different CRT schedules, weekly compared to every-3-weeks platinum-based regimens resulted in similar outcomes and less renal toxicity. The benefit of weekly platinum chemotherapy lies in the ability to titrate the dose to avoid severe acute toxicity. In our series, weekly platinum regimens were more often used in older patients or those with more comorbidity, indicating a potential selection bias. Based on prior data demonstrating improved disease outcomes in younger patients and those with less comorbidity [21, 22], we would expect the selection bias present in our series to result in worse outcomes for patients treated with weekly chemotherapy. However, weekly chemotherapy was similarly efficacious for patients with these worse prognostic features. Additionally, patients treated with weekly chemotherapy had significantly fewer AKI events compared to those receiving an every-3-weeks regimen. Thus, even with more adverse features, patients treated with weekly platinum-based CRT had similar disease control and survival rates with less toxicity.

In our study, similar rates of treatment compliance likely facilitated similar LRC and OS rates for weekly and every-3-weeks chemotherapy that were comparable to previously reported outcomes [2, 3]. Previously, it has been suggested that a weekly chemotherapy may be inferior to every-3-week chemotherapy as it is less likely to achieve cumulative doses ≥ 200 mg/m^2^ for cisplatin or ≥5 weekly cycles [23, 24]. Tsan et al. demonstrated that only 62.5% of patients treated with weekly cisplatin achieved cumulative doses of ≥ 200 mg/m^2^ compared to 88.5% of patients receiving every-3-weeks chemotherapy [24]. Similarly, previous retrospective studies reported that 58.5–71.0% of patients receiving weekly cisplatin achieved 5 or more weekly chemotherapy cycles resulting in cumulative doses ≥ 200 mg/m^2^ [23, 25, 26]. By contrast, trials using every-3 weeks cisplatin achieved cumulative doses of ≥ 200 mg/m^2^ in 79–84% of patients [2, 4]. Treatment compliance in our study is more compatible with prior trials using every-3-weeks chemotherapy, as 78.1% of patients treated in our study received at least 5 weekly chemotherapy cycles. In our study, patients in the every-3-weeks cisplatin group received a median cumulative dose of 200 mg/m^2^. However, we cannot adequately report the cumulative dose for patients treated with weekly chemotherapy as the majority of these patients were treated with carboplatin typically at an AUC of 1.5. Nevertheless, the number of patients receiving weekly chemotherapy cycles was greater in our study than previously reported and may partially account for similar efficacy between different chemotherapy schedules.

Furthermore, we observed that fewer patients experienced AKI when treated with weekly platinum regimens compared to every-3-weeks. While we did not observe significant differences in other acute or late toxicities, nephrotoxicity remains a dose-limiting complication of platinum-based chemotherapies. Our rate of AKI approached 50% with every-3-weeks chemotherapy and appears substantially higher than previously reported rates of severe acute nephrotoxicity. However, this discrepancy lies in the use of different definitions for clinically meaningful renal toxicity. Previous trials using every-3-weeks cisplatin which report grade ≥3 nephrotoxicity rates of 4.1–8.4% [2, 4, 27] use clinical criteria for renal failure to define such acute severe nephrotoxicity, including the need for dialysis and/or Cr elevations greater than 3.3–3.9 mg/dL among others. However, these criteria for renal failure likely underestimate small but clinically relevant renal insults. In our study, a Cr rise of ≥ 0.3 mg/dL was used per the Acute Kidney Injury Network definition of AKI [19] and has been associated with a 4.1-fold increased risk of mortality [20]. Furthermore, relative changes in Cr, rather than absolute rises, likely capture changes in renal function that better reflect individual differences in body mass. Thus, compared to every-3-weeks platinum, we observed that weekly platinum regimens were associated with fewer renal injuries that may not constitute renal failure but potentially impact patient survival.

Our study is limited by its retrospective nature and heterogeneous population. In this study, patients receiving weekly chemotherapy were more likely to be treated in the post-operative setting and receive carboplatin, whereas patients receiving every-3-weeks chemotherapy were more likely to be treated in the definitive setting and receive cisplatin. Previous reports including a Hellenic Cooperative Group Trial by Fountzilas et al. and a more recent study by Rades et al. have suggested that carboplatin-based CRT may be inferior to cisplatin-based CRT [28, 29]. However, Fountzilas et al. was not directly powered to compare cisplatin to carboplatin and Rades et al. used a non-standard every-four-weeks chemotherapy administration schedule. Since we did not observe differences in outcomes between cisplatin and carboplatin, our results suggest that weekly carboplatin-based CRT has similar efficacy as CRT with every-3-weeks cisplatin. Though our sample size may be insufficient to detect small differences in outcomes, treatment decisions balancing potentially small differences in disease control with larger differences in toxicity may still find CRT with weekly carboplatin favorable. The median follow-up of our study sample may also limit conclusions on control and survival that can be drawn between the groups. Nevertheless, our results were sufficient to observe less renal toxicity and less hospitalizations in patients treated with weekly platinum-based regimens. Ultimately, the use of weekly platinum-based concomitant chemotherapy with RT for the treatment of locally advanced HNSCC awaits prospective validation. Additionally, the comparative efficacy and toxicity of platinum chemotherapy versus epidermal growth factor receptor (EGFR) inhibitors, namely cetuximab, as radiosensitizers remains an area of great interest currently being studied in the RTOG 1016 and TROG 12.01 trials. Lastly, the low level of HPV-positivity in this study, which is consistent with prior results from our institution [30], limits the generalizability of our results to populations with higher proportions of HPV-positivity.

## Conclusions

In conclusion, our cohort of HNSCC patients treated with definitive CRT had similar disease control with either every-3-weeks or weekly administration of platinum chemotherapy. By contrast, acute renal toxicity was significantly less in patients receiving weekly chemotherapy. As weekly administration of chemotherapy in this setting awaits prospective validation, we must critically assess the balance between treatment efficacy and toxicity in the management of patients with locally advanced HNSCC.

## Additional file

Additional file 1: Table S1. De-identified patient and treatment characteristics. (XLSX 63 kb)

## Abbreviations

AKI: acute kidney injury
Cr: creatinine
CRT: chemoradiation
HNSCC: head and neck squamous cell carcinoma
KPS: Karnofsky performance status
LRC: locoregional control
MVA: multivariate analysis
OS: overall survival
PFS: progression-free survival
RT: radiotherapy
UVA: univariate analysis
